# Identification and Adoption of Themes in The Big Bang Theory Sitcom to Foster Academic Cultural Competencies of Doctoral Students in English for Academic Conversation Classroom

**DOI:** 10.3389/fpsyg.2021.699662

**Published:** 2021-09-09

**Authors:** Olusiji Lasekan

**Affiliations:** Departamento de Educación Media, Universidad Católica de Temuco, Temuco, Chile

**Keywords:** Ph.D. competencies, doctoral students, The Big Bang Theory, thematic learning, reflective practice

## Abstract

The primary purpose of this study is to identify academic cultural themes in the popular television sitcom The Big Bang Theory in order to enhance doctoral students’ awareness and acquisition of Ph.D. competencies through formalized and explicit instruction. The secondary aim is to assess the impact of the selected academic themes on doctoral students’ acquisition of Ph.D. competencies in an English for Academic Conversation (EAC) classroom. Drawing upon the concept of thematic learning instruction, a qualitative research method involving six clusters of Ph.D. competency reference framework developed by Durette and others was adopted to identify the academic cultural themes depicted by the sitcom’s main characters. This is followed by evaluating the effectiveness of an EAC course in fostering learners’ Ph.D. competencies using selected identified academic cultural themes. The result showed that the sitcom’s main characters demonstrate the personal and professional skills commonly possessed by a competent academic as an individual or group. This is evidenced as all the thirty-four identified skills traverse the six clusters of Ph.D. competencies devised by Durette and colleagues in 2016. Also, the impact assessment results revealed that the course fostered learners’ Ph.D. competencies as they shared knowledge, past experience, and action plans of every selected academic theme. This work contributes to existing knowledge of doctoral competencies vital to promoting competency-based Ph.D. programs in higher education.

## Introduction

Academic culture can be defined as shared attitudes, values, and behaviors of lecturers, researchers, and scholars in the university’s setting (Brick, 2020). This term is characterized with academic mindset, spirits, knowledge, appearance, and atmosphere, which are all aspects of the term (Shen and Tian, 2012). To apply this concept, numerous researchers have investigated academic cultural competencies, also known as Ph.D. competencies (Durette et al., 2016; Verderame et al., 2018). These competencies are advanced skills required to extend frontiers of knowledge and function effectively in academia (Bogle et al., 2010). Extensive implications of Ph.D. competency studies include curriculum mapping and program enhancement plans (Verderame et al., 2018). Graduate programs need this curriculum framework to develop learning objectives and outcome assessments that educational accrediting agencies commonly require. Since these skills are critical for future academics’ survival in academia, their integration into the curriculum should be considered essential in every doctoral program.

Many researchers have highlighted the skills and qualities Ph.D. students should possess by the end of their doctoral studies (Durette et al., 2016; Verderame et al., 2018). Durette et al. (2016) developed the most up-to-date and comprehensive Ph.D. competency framework. It consists of 111 Ph.D. competencies organized into six main categories (knowledge and specialized technical skills, formalized transferable competencies, unformalized transferable competencies, disposition, behavior, and meta-competencies) (Durette et al., 2016). Ph.D. programs around the world are already incorporating some of the Ph.D. competencies developed by Durette et al. (2016). While a doctoral program may require the students to acquire cognitive capacity, task-orientedness, and interpersonal skills by the end of the program, another program may require research skills and knowledge, communication, professional development, leadership, and management as their clusters of competencies. Thus, the Ph.D. Competencies Reference Framework developed by Durette et al. (2016) can be described as a reliable guide that can help to create a competency-based Ph.D. program.

Movies arouse interest by depicting reality (Tan, 2018). According to Tan, interest is the emotion required to establish a connection with, focus on, and perceive specific story-world events. Blasco et al. (2015) support this assertion by stating that a movie’s narrative impacts the viewer’s affective domains to stimulate learner reflection through a heightening of emotions. The application of its effect in the classroom involves identifying themes in a movie that can stimulate discussion and reflection, which are critical for skill or cultural acquisition. This is based on the argument that evaluation of competence depends on the learner’s ability to self-reflect and self-regulate on the skill. It is more accurate to say that acquiring skills is a process rather than an educational product, such as exams or professional reports (Lombardi, 2008; Lewis et al., 2011). The effectiveness of this thematic learning approach has also been demonstrated in different classroom designs to foster professional competence (Lalor et al., 2015), literacy competence (McLean, 2017) and diagnostic competence among medical doctors (Mamede et al., 2012).

Television series have developed into an excellent medium for transmitting knowledge and ideas and enhancing formal and informal learning (Ryan and Townsend, 2010; Lacko, 2011; Konus, 2020). While television show such as “Friends” has been used to improve learners’ English skills (Konus, 2020), the study of Lacko (2011) revealed the beneficial effect of medical dramas such as Grey’s Anatomy on medical doctors’ practice. For teachers, their representation on TV shows has been identified as a critical instrument for promoting thematic learning among preservice teachers and, as a result, developing their educational philosophy as well as their professional identities and instructional practices (Ryan and Townsend, 2010). Hence, it is thought that a TV show that portrayed the life of academics can be used as a thematic instructional tool to foster academic cultural competence in doctoral programs.

The Big Bang Theory (TBBT) is an American situation comedy (sitcom) that aired its first episode on September 24, 2007 and ended on May 16, 2019. It depicts scientific accuracy and up-to-date science information (Hewitt, 2009), mainly focusing on scientists’ social lives (Thomas, 2010). It revolves around male scientists (Sheldon, Leonard, Howard, and Rajesh) and a female actress/waitress, Penny. In addition, three female scientists (Leslie, Bernadette, and Amy) that have appeared in different seasons are also key characters in the sitcom. The educational and informative efficacy of the show has been demonstrated in several studies. Several educators have also used scenes from the show to teach various science concepts (Korek, 2011; Follert, 2015), economics (Tierney et al., 2015), and oral English communication (Palu, 2016). Therefore, since the sitcom depicts academics’ lives, it is argued that it can serve as a thematic instructional tool to foster doctoral students’ competences.

Considering the importance of a competency-based Ph.D. program, many graduate programs are yet to implement this holistic approach of training Ph.D. candidates. This can be attributed to a widely held view that only knowledge and research ability skill of a chosen field can be formalized, while the rest of the established competencies can be acquired informally (Larkin and Morris, 2015). This has contributed to many doctoral students and early career scientists leaving academia due to their lack of workplace survival skills (Etzkowitz et al., 1994). Therefore, creating a model that can formalize all established competencies’ learning and acquisition is critical. This will help graduate programs in providing explicit instruction on these essential competencies (Fischer and Zigmond, 1998).

Against this background, this study seeks to identify academic cultural themes in TBBT that correspond to Ph.D. competencies proposed by Durette et al. (2016) reference framework, use them to foster academic cultural competence in an EAC classroom, and assess the impact of the course on doctoral students’ learning outcomes. Thus, this paper begins with the design of the theoretical and conceptual framework, followed by reviewing the literature, methodology, results and discussion, and the conclusion, which gives a summary and critiques of the findings. Throughout this paper, the term academic cultural competencies will be used interchangeably with Ph.D. competencies.

## Theoretical and Conceptual Framework

The purpose of this study is to identify academic cultural themes in TBBT and to incorporate them into an EAC course in order to help postgraduate students develop their Ph.D. competencies. Thematic learning is a method of instruction that entails the adoption and application of themes in order to foster an active, interesting, and meaningful learning environment (Min et al., 2012). It is predicated on the premise that students acquire knowledge or skills more efficiently when they learn coherently and holistically and can relate what they learn to their surroundings and real-world examples (Mirjalili et al., 2012). The effectiveness of this approach in developing any skill in learners is that it enables teachers to present a problem within the context of a chosen theme in order to encourage reflection and the provision of a solution (Davies and Shankar-Brown, 2011). However, the process of thematic learning instruction is frequently defined as identifying and integrating themes into classroom activities. However, the instructional procedure for this integrative practice is determined by the teaching objective and the integration tool chosen.

Movie clip integration is an effective way to introduce a new theme or difficult concept into a lesson plan. It contextualizes any given subject in order to facilitate a critical discussion (Arguel and Jamet, 2009). In other words, the use of meaningful video clips in the classroom may be most appropriate for introductory courses, introducing complex topics in general, lower-achieving students, and visual/spatial learners (Berk, 2009). The effectiveness of this approach to facilitate language learning is based on a cognitive theory of multimedia learning proposed by Mayer and Moreno (1998). It asserts that when information is presented in both text and graphics, rather than just text, deeper learning can occur. It is predicated on the assumption of two distinct learning modes: auditory and visual, which are channels used to store data in working memory.

Dialogic learning is one of the most effective methods for developing students’ knowledge on a subject matter. It entails harnessing the power of conversation or discussion in the classroom to promote idea sharing to master a specific disciplinary knowledge and advance students’ learning and comprehension of any topic or theme (Haneda, 2017). In other words, learners take on the role of protagonists in their own learning process when they engage in dialogs with peers who assist them in attaining higher levels of thinking, reasoning, and comprehension than they could achieve on their own (Flecha, 2000). Additionally, it alters classroom dynamics by rebalancing the traditional power dynamic between teachers and students (Teo, 2019). This approach is founded on democratic values-based dialog, in which students collaborate to gain understanding and complete tasks, progressing in their thinking and reasoning (García-Carrión et al., 2020).

Reflective practice can be defined as the process of turning thoughtful practice into a potential learning situation (Kember et al., 2000). According to Johns (1995), the purpose of this practice is to facilitate the interpretation of a process or experience. It is used to improve understanding of relatively complex or unstructured ideas and is largely based on the reprocessing of previously acquired knowledge, understanding, and possibly emotions (Chelliah and Arumugam, 2012). Reflective practice has been used in studies to both promote and justify skill acquisition (Lalor et al., 2015; Lutz et al., 2016). Lynch and Clinton (2020) state that good evidence of competency should demonstrate that a learner understands how to apply knowledge, skills, and experience through reflection. The process begins with an assessment of their knowledge and experience with the concept, followed by a determination of their willingness to continue applying and expanding their understanding of the theme (Campinha-Bacote, 2002). Thus, the acquisition of competencies is not limited to test results, reports, evaluations, certificates, or licenses (Boud et al., 1985).

**FIGURE 1:**
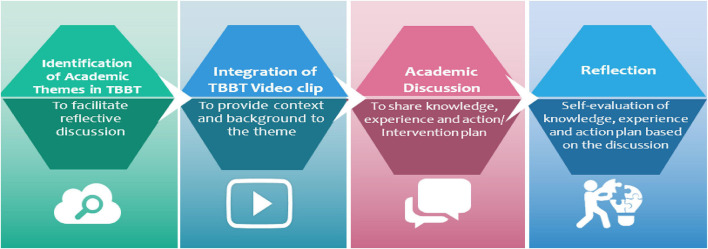
Conceptual framework on the process of academic cultural competence acquisition.

On the basis of the foregoing concepts, a model (see Figure 1) for fostering academic cultural competence was constructed. The model details are as follows:

It is predicated on the premise that popular culture, such as film, reflects society’s values, norms, and beliefs. Thus, by embracing thematic learning as an instructional strategy, TBBT, a popular culture show depicting scientists’ lives, can be used to facilitate discussion and reflection among doctoral students about the academic skills, values, ethos and standards. The process of acquiring Ph.D. competencies entails identifying which sitcom academic cultural themes correspond to the Ph.D. competencies highlighted by Durette et al. (2016). Following that, the video clips are integrated into the classroom to provide context and knowledge background for the discussion. Then, using the dialogic learning concept, whereby a discussion is facilitated in order to develop general information, experience, and knowledge about the theme. This step is carried out with the assistance of a prompt as a task-based activity. This entails developing and administering a list of questions [the Question Prompt List (QPL)] designed to facilitate discussion between two learners to generate shared general knowledge, information, and experience. Finally, Ph.D. competency acquisition is evaluated based on the learner’s ability to reflect on personal knowledge and prior experience related to the topic presented in the immediate discussion and express personal action plans related to the theme.

## Literature Review

Ph.D. competency framework adoption in graduate programs has seen a steady rise over the past few years. While the number and types of skills adopted for their framework may vary from discipline to discipline, the goal is to build a framework that prepares professionals and research scholars to function effectively in academia or industry. To foster the training of these skills, a study recommended researchers’ movement across institutions/nations, supporting graduate students’ mobility (conferences, student exchanges, workshops, and joint and exchange programs), and appreciation for diversity through films, readings, and discussions in courses (Niemczyk, 2018). In the author’s opinion, the following problems could impede the recommendations’ implementation in most institutions: This includes a lack of funding and resources to support students’ participation in conferences, creating international partnerships, promoting collaborations with research-oriented solid institutions, and so on. Also, recent research argued that the number of academic cultural competencies acquired depends on doctoral mode of education. Lindner et al. (2001) demonstrated that on-campus students had a higher perceived competency than distance learners. In sum, fostering doctoral competencies in higher education is challenging and it depends on the program’s mode. The gap in the literature is a framework that can help formalize every aspect of academic cultural competencies.

The Big Bang Theory has been the target of numerous educational studies. Foremost, a study focused on how the sitcom portrays the life of a group of scientists that is more diverse in terms of gender, ethnicity, and most importantly disciplinary focus than what is commonly seen on television (Weitekamp, 2017). The author highlights how the characters demonstrate scientific authenticity, brainstorm ideas, and academic working structure. This discussion metamorphosed into how the audience perceives the show. For example a publication is aimed at strengthening the media’s role in communicating science (Li, 2016), and the results show that the sitcom stimulates many audiences to discover more about the scientific information presented in the show. Also, the viewers are willing to learn different facets of science when watching entertainment television. Moreover, recent publications have adopted the sitcom as a resource to facilitate learning in several disciplines. Geerling et al. (2018) use clips of the show to teach numerous concepts of economics. Another study analyzes the interruptions on the show to teach how to ask and give an opinion (Annisa, 2016). Lastly, TBBT is used in the field of English as a Foreign Language to develop teachers of English’s knowledge of American culture, which is critical for their students’ English learning (Lee, 2016).

Taking these considerations into account, the purpose of this study is to identify academic cultural themes in TBBT, to align these themes with Durette et al. (2016) Ph.D. competencies, and use the themes to foster doctoral academic cultural competence in the EAC classroom. The research was motivated by the following questions:

1. What are the identified academic cultural themes in TBBT that correspond to Ph.D. competencies developed by Durette et al. (2016)?
2. What is the effectiveness of the EAC course in fostering learners’ Ph.D. competencies using selected identified academic cultural themes?

## Research Design

**FIGURE 2:**
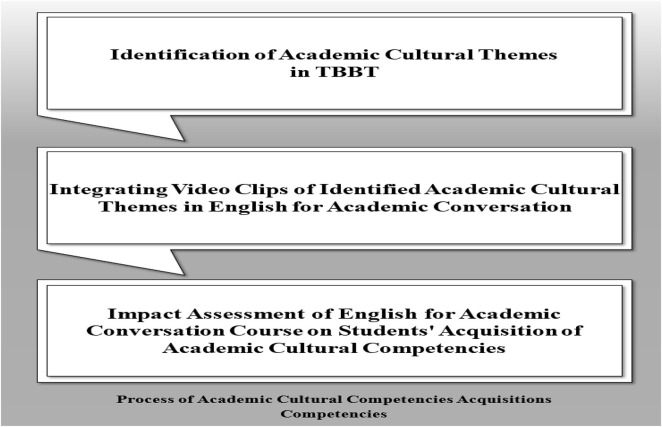
Research design on the process of academic cultural competencies acquisition.

This paper seeks to identify academic cultural themes in TBBT and to foster Ph.D. competencies among doctoral students. The research design is based on philosophical approach. That is, researcher’s assumptions about how data should be gathered, analyzed, and adopted (Collins, 2010). Figure 2 shows that the study design involves adopting Durette et al. (2016) Ph.D. competency framework, using video clips of the various themes to foster Ph.D. competency, and assessing the impact of the themes in the videos on students’ learning outcomes.

## Themes Identification

### Source of Data for Themes Identification and Analysis

The data for this analysis was sourced from TBBT episodes. Chuck Lorre, Bill Prady, and Steven Molaro all served as executive producers on the series. The show debuted on CBS on September 24, 2007, and concluded after 279 episodes over 12 seasons. Leonard and Sheldon share an apartment with Penny, who is an aspiring actress. They are friends and coworkers to Howard and Raj, who are aerospace engineers and astrophysicists at Caltech. Also starring is microbiologist Bernadette Rostenkowski and neuroscientist, Amy Farrah Fowler. Other supporting characters include an experimental physicist, Leslie Winkle and comic book store owner, Stuart Bloom.

### Instrumentation for Themes Identification and Analysis

Durette et al. (2016) built the Ph.D. Competencies Reference Framework, a recently updated comprehensive competency framework which provides enough detail and accuracy to meet the skill needs of a Ph.D. student. The researchers utilize a two-tiered approach. Normalization is the first step (Arampatzis et al., 2000). Semantically similar terms are mapped to a single canonical representative word. A combination of morphological normalization (Arampatzis et al., 2000). The scholars classify all of the 16,000 expressions or words linked to competencies into 500 semantically similar clusters. Human resource experts analyzed their clusters to learn about their importance and accuracy. When needed, clusters were broken into smaller clusters. Second, clusters were arranged into increasingly finer levels of precision in the data. Using a bottom-up approach, their human resource experts used the grounded theory framework in the procedure (Strauss and Corbin, 1998). Identifying similar competencies within a cluster defined lower-level definitions (though semantically distinct). The process was repeated until no more significant groupings appeared. Participants’ outputs and competencies are communicated and coordinated. The study identified 111 terminal nodes (leaves) organized into six main categories and three hierarchical levels. Those six categories, which emerged from the clustering process, are:

• Knowledge and Technical Skills: These are well-known techniques and knowledge that must be learned in particular or several disciplines.
• Transferable Competencies that can be Formalized: These are skills that can be incorporated into various clinical environments. Communication (oral and written), creativity management, and scientific monitoring are all core competencies in this group as well as project management, time management and planning, and languages.
• Transferable Competencies that cannot be Formalized: These are skills that can be applied to any field but are not typically acquired by formalized Ph.D. studies. Cognitive ability, problem-solving skills, and teamwork skills are all core competencies in this group.
• Dispositions: These are critical in defining one’s work attitudes and ethics, complementing the other competencies. Rigidity, critical thought, imagination, and autonomy are the central competencies in this group.
• Behaviors: These competencies provide what are known as “soft skills,” such as social and interpersonal abilities. Perseverance is the category’s main competency, but it also involves empathy and diplomacy.
• Meta-competencies: Meta-competencies are important for sustaining and increasing one’s pool of competencies over time and allowing efficient use of other competencies in professional settings. In this category, a Ph.D.’s main metacompetency is adaptability.

The competency system strikes the right balance between structure and versatility. According to Durette et al. (2016), it is prescriptive because it identifies shared goals and principles, thus assisting in developing appropriate instructional practices. The tool was initially designed to evaluate the competencies of French academics.

### Data Collection Procedure for Themes Identification

Observation and recording are the data collection strategies used. The descriptive analysis technique is used to identify and analyze the academic cultural themes in TBBT’s data. Every episode from Seasons 1 through 12 were hand-picked for examination. In Manganello et al. (2008), seven episodes from a TV season were needed to produce character-based findings, however, this research is significantly more thorough and organized than this advice, more similar to Ye and Ward (2010). TBBT was chosen because it is the only existing TV show that depicts researchers’ character. In comparison to a movie, a TV series has more complex content that viewers can analyze, which is why the sitcom is preferred over a movie. The investigator reviewed each important scene many times. After watching the full episode, the researcher took notes on which individual interactions satisfied the evidentiary competencies in the framework. The researcher transcribed the dialog during the second screening. After the final view, the researcher recorded any subtle verbal intonation and any non-verbal images. To code and match key interactions, competencies in each cluster were used.

### Content and Thematic Analysis of TBBT

This study utilized a qualitative content analysis research approach and analyzed data using an adapted procedure of Fraenkel and Wallen (2009) and Lacko (2011). In a recent research, content analysis was used to evaluate website content (Lasekan et al., 2020). The first step in conducting a content analysis is establishing research objectives. The researcher’s primary objective in this study was to portray characters in TBBT as competent researchers through the use of Durette et al. (2016) Ph.D. competencies as a checklist for identifying academic cultural themes. The central unit of investigation is the analysis of the characters’ interactions. The interaction must have lasted at least 5 seconds to qualify as critical and should be critical for the story’s advancement. The significance assessments are based on analysis units’ rationale of Ye and Ward (2010). The data analysis techniques used in this study were manifest and latent content analysis. It is combined with qualitative theme analysis elements. In other words, to categorize components of an established communication measure (the Common Ground Instrument; Lacko, 2011), the researcher began with content analysis and then moved on to thematic analysis in order to examine entire themes and ideas. The description of the analysis is included below.

Manifest Content Analysis – It refers to “that which is specifically articulated” (Clarke and Binns, 2006, p. 41) or “those physically present and countable elements” (Clarke and Binns, 2006, p. 41). This particular application of the deductive principle has two advantages (Lacko, 2011). To begin, the manifest content analysis assesses unstructured data in accordance with Berg (1989) and Krippendorf (1980). As a result, analyzing the manifest content of these experiences produces a more exhaustive list of themes than evaluating them using a standardized instrument. Finally, content analysis can be used to explain the content patterns of communication (Berelson, 1952).

Latent Content Analysis – It refers to a text’s “underlying and implied context” (Neuman, 2000, p. 296). Clarke and Binns (2006) define latent analysis as an inductive method of reasoning, which means that comprehension is derived from data rather than from preconceived theories (Krueger and Casey, 2000). This induction enables researchers to identify previously unknown themes or factors that they would not have discovered if they had preconceived ideas about what to look for Lacko (2011).

Using the framework of Durette et al. (2016), explicit categories for show analysis were developed in this study. This entails interpreting and connecting with adapted evidential competencies. This is followed by the categorization of manifest and latent themes according to the length of the video clips that correspond to the episodes.

### Thematic Analysis in Qualitative Research

Along with the analysis of manifest and latent content, this study incorporated elements of a qualitative theme analysis. This is aimed at using a more robust and rigorous form of qualitative analytical methods to assess a television show. While using a second rater helps provide credibility to a thematic analysis, adopting a single coding technique is also acceptable and reliable if the coder is knowledgeable about the subject (Campbell et al., 2013). The investigator’s knowledge is based on the fact that he has followed every episode of the series for the past 12 years. However, as a lone researcher, several steps were taken to strengthen the reliability of the analysis. This includes sharing with fellow academics who are strong followers of TBBT about the coding and analysis as the investigator progressed through them. This concept, called “member checking,” involves sharing coded field note excerpts and discussing any “doubt and dilemmas” about coding and analysis generating peer support. This helps in reconciling views regarding the rating of themes categories in progress (Burant et al., 2007). Discussion provides an opportunity to articulate internal thinking processes and presents windows of opportunity for clarifying emergent ideas and possibly making new insights about the data.

## Integration of Video Clips

### English for Academic Conversation

This is an 8-week course. Each class lasts 90 minutes; each week, the class discusses various topics that correspond to identified academic cultural themes in TBBT. The course was developed in response to a need to strengthen students’ research capacity, academic job prospects, international mobility, and scientific productivity, all of which are anchored on the oral dissemination of academic knowledge in English. Thus, the course’s primary objective is to provide an environment in which students can discuss and converse in English about their research topic and experience and practice their English conversation skills by discussing issues relevant to researchers’ lives. The process entails facilitating each topic by setting the stage for an in-depth discussion. The objective is to develop an understanding of academic culture that will aid them in achieving their academic career goals.

With respect to the selection of video corresponding to a theme, as previously stated, EAC is an 8-week online course. Altogether, data from six class meetings are available for this study. The selected videos cover a range of topics, including focusing skill in the Dispositions cluster, research funding and public speaking in the Transferable Competencies that can be Formalized cluster, teaching and research ability in the Knowledge and Technical Skills cluster, and overthinking in the Transferable Competencies that cannot be Formalized cluster. Each class begins with a task that serves as a pre-discussion (10–15 min). The topic is introduced to the students at this point. Through question and answer, the instructor and students negotiate the theme’s vocabulary. After that, the video clip corresponding to the theme is played to provide context and background information, which are critical for facilitating discussion. Many of the clips are between 3 and 5 min long. Because the comprehensibility of the video clip is critical for the discussion, and some students have limited level of English listening skills, students are shown video clips that have been dubbed in Spanish.

### Facilitation of Academic Conversation

The adoption of discussion stems from the importance of stimulating interlocutors’ thinking, comprehension, and reflection. This type of pair or group discussion is possible when each participant possesses information, experience, and knowledge that others lack (Rees, 2005). As a result, an interlocutor using the functional language must bridge an information, experience, and knowledge gap. To close this gap, a prompt is used as a task-based activity to facilitate the discussion. The task-based activity, also known as a QPL, is used in the medical field to facilitate communication between patients and physicians and to encourage active participation in physician-patient discussions (Eggly et al., 2013). The prompts (included in Supplementary Appendix Table B) are designed to elicit knowledge, information, prior experience, and the development of objectives related to the theme. The procedure begins with the distribution of a list of Question prompts to a pair of students for discussion. Breakout classrooms on Zoom were used to facilitate this online discussion. They are online classrooms that are used to create spaces for small groups of learners to “meet,” work, and collaborate (Kohnke and Moorhouse, 2020). The students were instructed on the activity’s significance to express their opinions and thus achieve the desired learning outcome. Throughout the discussion, the instructor rotates between classrooms, spending 4–5 min to observe and assist students who are having difficulty with English vocabulary to establish effective English communication. As a result, the instructor enters the room with the camera turned off and the microphone set to mute in order to avoid any unnecessary interruption or intervention.

### Self-Reflective Task

During the final section of the session, students are required to reflect on their self-awareness, personal experience, and personal plan objectives as a result of the immediate discussion. The purpose of this task is to determine the discussion’s learning outcomes. This process began upon the students’ return from the breakout classrooms to the main session classroom. Students are then asked to share what they’ve learned in class, including other partners’ new information, knowledge, and perspectives, as well as comments on their oral fluency and future action and intervention on the theme.

## Impact Assessment

### Participants in the Study

This study included eight participants. They all enrolled in EAC. They are all postgraduate students from a variety of faculties, including pure sciences, social sciences, and the humanities. Their ages range from 26 to 35. While five are pursuing doctoral degrees, three are pursuing master’s degrees with a research major. Each is in a different stage of their postgraduate studies. They are native Spanish speakers with an intermediate level of English and they aspire to pursue academic careers in the future.

### Data Collection and Analysis Procedure

A phenomenological qualitative approach was used to add substantive depth to the body of knowledge on this subject. This approach gather data from individuals who have direct experience with the phenomena under investigation (Patton, 2015). This study collected data using methods commonly used in observational research and qualitative research, such as interviews. The qualitative design method was chosen to examine the effect of video clips-based academic discussion on students’ academic cultural competence. The impact assessment is conducted in two stages: the first stage assesses the impact of each session on learners’ Ph.D. competence acquisition. The second stage is to ascertain the learners’ attitudes toward the EAC course and TBBT show. Acquiring Ph.D. competence is predicated on the tenet of good competence evidence (Lynch and Clinton, 2020). This is the capacity of a learner to construct knowledge about a competence, connect his or her experience to the competence, and then express future action and intervention plans using reflection. Two researchers independently analyzed the six session recordings (with topics of focusing, overthinking, research funding, teaching, research skill, and public speaking). Separate files containing recordings and transcriptions were created for each of these themes. The researchers then coded the transcriptions and met to discuss their notes and observations. Responses to QPL are coded according to three categories: knowledge, experience, and future action/intervention plan. Each code is operationally defined and was refined and agreed upon prior to analyzing the recordings by both raters. While one of the raters is an expert at incorporating reflection principles into the classroom, the other is inexperienced. A measure of agreement was used to determine the reliability of each pair of raters for each reflection element (Plack et al., 2005). Percent agreement is one of the most straightforward and intuitive measures of inter-rater reliability. The level of agreement between the raters was determined using Kappa’s statistic value of agreement, which ranges from zero (no agreement) to greater than 90% (almost perfect agreement) (McHugh, 2012). Only simple agreements with 80% and above were considered in this study. For instance, raters were asked to indicate their level of agreement with the definition of self-knowledge, past experience and action plan as described by Lin and Jain (2018). Self-knowledge is the capacity to identify and explore one’s own assumptions, values, and beliefs. Past experience is defined as the ability of the participants to relate the concept or skill to a recall past event or incident in which they learn from. The ability to describe the strategies used to sustain and acquire the skill and acknowledge and begin working with the feelings associated with the skill and improve understanding of one’s knowledge regarding the skill is referred to as an action or intervention plan.

The researcher served as an interviewer, observer, data processor, recorder, and analyzer for the participants in this second stage of the impact assessment analysis by evaluating their perception of the course. Ethical issues were a top priority throughout the study. The researcher informed participants that their participation was entirely voluntary and confidential. To protect confidentiality and privacy, proper precautions were taken during data collection and analysis. Prior to the research study, participants were informed about the study’s components, what their participation would entail, and given the option to participate. To understand abstract cognition phenomena, qualitative studies should be conducted in a natural environment (Creswell, 2014). Thus, the participants’ observations were conducted during regular class time by video recording the whole class. At the end of the course in 2020, all eight participants were recruited for interviews. The combination of participant interviews and observation enabled depth and inferences that would not have been possible with a single data collection process (Patton, 2015). The interview section focuses on general reactions to the video clips and the class. Several questions address the effect of video clips on academic discussion, the relevance of the video to the given topic, understanding how Ph.D. competencies are acquired after watching the clips, and how it motivates them to join academia in the future. Other questions include their desire to watch the show after completing the course and their suggestions for improving the course. The information gathered from the video recording for class observation was transcribed. As a result, the researcher has data that includes interview transcripts and class responses. This data was analyzed by coding it, finding patterns in it, categorizing the patterns, and finally developing a method for categorizing it (Patton, 2015). Before categorizing the data, it was analyzed and compared using the constant comparative approach. These categories were used to create and apply patterns to help answer research questions. Direct quotes and interview details were included in the findings report to add insight and clarification to the study results.

## Results

### TBBT Content Analysis

The first research question sought to identify academic cultural themes in TBBT. Themes that corresponded to the 111 Ph.D. competencies developed by Durette et al. (2016) were identified using content and thematic analysis. Out of 111 Ph.D. competencies, thirty-four skills that spread across the clusters of the Ph.D. competencies framework were identified. Supplementary Appendix Table A contains the complete version of the identified themes along with the corresponding Ph.D. competencies. The following are explanations for these themes.

### Knowledge and Technical Skills

This competency cluster is known as techniques and knowledge that one develops in one or more disciplines. In other words, it can be both monodisciplinary and multidisciplinary.

#### Monodisciplinary

By revealing the characters’ accomplishments, TBBT portrayed all of the scientists in the show as top experts in their field. Sheldon, the main character, is a theoretical physicist who received a grant to study String Theory at the North Pole (S2, E23) and went on to win a Nobel Prize (S12, E24). Leonard is an experimental physicist who won a grant to visit the CERN supercollider in Switzerland (S3, E15) and work with Stephen Hawkings (S6, E24). Raj, an astrophysicist, is portrayed as the best in his field when he is named one of People magazine’s “30 under 30 to watch” (S2, E4). Howard has a Master’s degree in Engineering and his most notable accomplishment was building a Wolowitz zero-gravity waste disposal system for NASA before becoming an astronaut (S5, E24). Amy’s character is described as a highly accomplished neuroscientist who received a grant from an Arabic philanthropist to fund her lab (S4, E15), won a summer research fellowship at Princeton University (S10, E23), and eventually won the Nobel Prize with Sheldon Cooper (S12, E24). Bernadette obtained a Ph.D. in microbiology and a lucrative position in a pharmaceutical company. She announced in S12, E13 that the FDA had approved the drug she had manufactured.

#### Pluridisciplinary

This is clearly demonstrated in S4, E20, when physicist, Sheldon and neurobiologist, Amy, spread a rumor to test meme theory. The fact that this is a social science concept outside their area of expertise demonstrates their pluridisciplinary skill and knowledge as a researcher.

#### Teaching

The show’s theme of teaching is very profound. The importance of teaching in academics is highlighted (S4, E14) when Sheldon’s students rated him poorly in terms of teaching. This is followed by various suggestions and exercises on how to improve his teaching ability from his friends. Sheldon’s poor teaching skill was once again demonstrated when he was unable to teach physics to Penny (S3, E10). Also a Master’s degree holder’s (Wolowitz) ability to teach a class assigned to a doctorate holder (Sheldon) is revealed in S8, E2.

#### Publication of Research Findings

This is yet another important academic theme in TBBT. Amy informs Sheldon of her publication in the prestigious Neuron Journal (S5, E12). Sheldon criticizes the work of his superior. He describes him as having written a series of popular books in which he reduced science’s great concepts to a series of anecdotes (S1, E4). Sheldon has an authorship dispute with Ramona, a postgraduate student, who helps him to focus on the research task (S2, E6). Sheldon and Leonard publish a paper based on Leonard’s idea, which is widely accepted with the exception of some insulting comments from one internet troll (S8, E14). Finally, Leonard and Sheldon were racing to publish their Superfluid Helium theory before the Swedish scientists publish their own findings (S9, E6).

### Transferable Competencies That Can Be Formalized

These are competencies that can be applied in professional contexts and learned through courses. Knowledge of the academic and industrial environments, professional conduct, communication skills, innovation management, languages, commercial skills, and administration management are all skills in this cluster.

#### Academic and Industrial Setting

In terms of academic knowledge, images in several scenes of the show depict a typical academic environment. The most common image in the show is of a laboratory. Leonard’s (S6, E5), Howard’s (S5, E16), and Amy’s laboratories (S5, E16) were all shown. Several scenes, such as the scientists’ offices, appear frequently in the show. Sheldon works as a theoretical physicist in an office with a whiteboard. The position of Raj as an astrophysicist was also revealed. The cafeteria is also frequently depicted in the show. It is a place where the characters have lunch, interact and discuss their research. Several campus events are shown, including science bowl (S1, E13), an award ceremony for Sheldon (S3, E18), a parking space issue on campus (S6, E9), and the tradition of showing new staff around campus on their first day (S1, E12). This exemplifies how the sitcom depicts a typical academic setting. Bernadette’s workplace can be used to describe a typical industrial setting. Her presence in both the lab and the office and her working relationship with Penny (S12, E17) sheds light on the life of a researcher in industry.

#### Professional Conduct

Several scenes demonstrate how to follow certain rules and regulations in academic settings. Firstly, Sheldon’s published work was retracted due to false data (S7, E10). This happened when his colleague informed Sheldon that the data provided to him at the North Pole was incorrect. Sheldon was also warned by human resources after using sexist language against his assistant (S6, E12). Also keeping the NASA project classified (S2, E22), and engaging in the unethical practice of purchasing helium on the black market for research purposes (S9, E6). In terms of safety, the scientists are shown using a portable safety shield in their respective labs throughout the sitcom.

#### Academic Communication Skill

There is a focus on the show’s word choice. In S4, E5, Sheldon and Amy are aware of the academic vocabulary they use in their normal conversation, possibly to demonstrate the academic English vocabulary commonly used by academics. Amy used the word “mellifluous” while Sheldon used the word “cornucopia”. Also, numerous suggestions on how to overcome fear of public speaking were given to Sheldon in S3,E8.

#### Information Technology Skill

Sheldon is portrayed as a character who is knowledgeable about information technology (IT). In S1, E16, while purchasing a gift in a store, he was preoccupied with advising and assisting other customers in selecting the appropriate computer accessories.

#### Innovation Management Skill

Research valorization is a management skill that entails using research results funded by the public for socioeconomic purposes (Bozeman, 2000). This theme is expressed in S4, E15 by emphasizing the importance of public and private donors’ contributions to research. The entire episode revolves around a research fundraising dinner, at which all of the invited scientists are expected to present their current work in order to secure funding. The audience also sees the university’s collaboration with several government agencies. Howard, for example, completes a NASA project (S5, E5) and works with Leonard and Sheldon to complete an airforce project for the United States military (S10, E2).

#### Project Management Ability

Learning how to manage a project is also important. In. S10, E3, Sheldon, Leonard, and Howard are assigned a task and given 2 months to complete it. They recognize the value of time management by devoting extra hours to the project. As a team leader, Howard effectively coordinates project management by requiring Sheldon to complete his portion of the task when they realize that completing the job in 2 months is not feasible. They report to the air force representative, who eventually extends their deadline of submission.

#### Languages

The Big Bang Theory demonstrates the characters’ enthusiasm for learning languages. Howards is seen teaching Sheldon Mandarin in S1, E17. This is also revealed when the male characters speak Klingon (S2, E1; S2, E7). This is due to their enthusiasm for Star Trek.

#### Commercial Skill

The characters are portrayed as more than just excellent scientists. They are also portrayed as characters who are well-versed in business and money management. Sheldon claims that he only spends 46.9% of his income and save the rest, which explains why he has enough money to loan Penny (S2, E14). In the S8, E4, Leonard suggests the idea of investing in Stuart’s comic book store and strategize how to compete with their rivals. In S9, E16, we learn how a scientist can commercialize their patent. For example, Howard decides to create a guidance system that can be patented.

#### Administrative Management

The show revealed how Sheldon had to hire an administrative assistant to demonstrate that administrative duties are also part of academic work (S6, E3).

#### Academic Categorization

The show provides insight into an academic hierarchical structure. When Sheldon, Raj, and Leonard compete for a tenured position in an American context, the audience understands the concept of “tenure” (S6, E20). Sheldon’s tenure track was revealed when he was promoted to the level of Junior Professor (S8, E2).

#### Imposter Syndrome

This is a psychological mindset that occurs when an individual believes their success is undeserved. That is, he or she is afraid of being exposed as a con artist (Hawley, 2019). Amy rants at Pemberton and Campbell, claiming that they are impostors and frauds, and that she and Sheldon are suffering from “imposter syndrome” (S12, E18).

### Transferable Skills That Cannot Be Formalized

They are competencies that can be applied in a professional setting but cannot be learned in a classroom setting. This includes lateral thinking, cognitive ability, collaborative skill, and leadership skill.

#### Lateral Thinking

It is used when the Howard space toilet fails (S2, E22). Sheldon is portrayed in the show as a character with lateral thinking skill. For example, it is very impressive in the way Sheldon recites the game of chance known as Rock, Paper, Scissors, Lizard, and Spock (S2, E8).

#### Cognitive Ability

Sheldon is aware of his superior cognitive ability. He believes he has an eidetic memory in S3, E5. In S4, E6, he demonstrated his long term memory by recalling a minute detail of an event. In terms of general knowledge, the character is also versatile in a variety of fields. He uses his chemistry knowledge to save Leonard’s life (S3, E22), and his bioluminescence knowledge to create a “Luminous Fish nightlight” (S1, E4). Furthermore, he used his geography knowledge to host his TV show, Fun with Flags (S1, E4). For innovation, Sheldon created his own game called research lab (S3, E7) and invented his own 3-player chess game (S4, E22).

#### Complex Problem Management

This is demonstrated when Sheldon and Penny collaborate to solve string theory (S11, E13). To be inspired, Sheldon solves a difficult physics question by focusing his mind on other tasks. The method has been shown to be effective. Thus, the process of solving a task-based problem without overthinking can be learned from Sheldon’s approach when he decided to work in a restaurant to solve the research problem (S3, E14).

#### Teamwork Skill

The importance of teamwork is demonstrated in the annual physics bowl competition (S1, E13). Sheldon is to be blamed for his team’s defeat in the competition because he refused to collaborate with his teammates.

#### Collaboration

It is a common theme in the show. Sheldon is seen at the North Pole working with other characters (S2, E23). Raj worked for Sheldon (S3, E4), Leonard, Howard, and Sheldon collaborate to work on a military project (S10, E2), and Amy and Sheldon collaborate to determine the exact time when the wave function collapses (S10, E19).

#### Leadership

Leonard’s ability to lead is critical because he is the glue that holds the characters in the show together. He was given the task of distributing funds in the show (S12, E7). He demonstrated his ability to delegate by directing his friends to search for Sheldon after he was reported missing (S7, E1). Throughout the show, Dr. Siebert, the president of Cal Tech, is also seen in a leadership role.

#### Behavior

Only seven of the thirty-four competencies listed by Durette et al. (2016) were identified under this cluster. ***Curiosity*** is one of the critical competencies. This is demonstrated by how the scientists searched for crickets in order to determine their species (S3, E2). The ability to ***dream*** is the second skill. Sheldon is always hopeful that one of his works will be awarded a Nobel Prize. He once told his friends that he would not invite them to his acceptance speech (S3, E1). Another popular topic is ***hygiene***. Sheldon demonstrates a sense of hygiene several times throughout the show, including using a baby wipe before eating (S2, E4) and Purell after touching a snake (S5, E7), as well as giving sanitizer to his friends before entering his house (S5, E7). Every character in the show displayed a sense of ***humor***. Sheldon demonstrates ***punctuality*** when he and other characters plan to see a movie. He insisted that they leave for the cinema sooner (S4, E8). Another competency in this cluster is ***service orientation*.** The university assigned Leonard, Sheldon, and Howard a social responsibility project. They choose to carry out an intervention task by encouraging young female high school students to pursue careers in science (S6, E18). All of the characters in the show demonstrate ***tolerance***. Sheldon acknowledges the longstanding patience of his friends in the last episode of the show (S12, E24).

#### Disposition

One of the competencies in this cluster is the ***focusing*** skill. In S8, E5, the scientists intended to innovate over the weekend, but they were unable to focus. They devise various strategies to aid concentration. Raj expresses one of a researcher’s key competencies in S4, E22. He claims to have an ***observation*** skill. “We are scientists, we observe everything,” he explained. Another skill is ***originality***. Sheldon always points out that Leonard’s research is based on someone else’s work (S3, E10). ***Accuracy*** is an additional skill. Scientists debate the scientific accuracy of events; in the Superman film, they argue about the speed at which Superman decelerates to save Lois Lane (S1, E2). Sheldon demonstrates ***persuasive*** skill when he begs Howard to show his article to Stephen Hawking (S5, E21).

#### Meta-Competencies

These are the skills to adapt one’s pool of competencies to various professional situations and contexts. **Adaptability**-Sheldon demonstrates how they adapt to all of the changes in his life. Firstly, Leonard and Penny’s engagement announcement distorts his attachment to his best friend; Secondly, an accidental fire destroys his favorite comic book store; and lastly, the university requires him to switch from string theory research to inflationary cosmology (S7, E24). Nonetheless, beginning with S8, E2, Sheldon was seen adapting to these changes.

### EAC Course Analysis

The study’s second objective is to investigate whether EAC can foster Ph.D. competencies acquisition in the classroom. This required determining the learners’ ability to acquire competencies, and then assessing their perception of the course. Six major themes were brought together, as can be seen in Table 1. Assessment of Ph.D. competencies acquisition is done by evaluating the learners’ knowledge, past experience, and action plan addressing a theme. The results show that the learners obtained all the skills assessed (overthinking, research funding, teaching, public speaking, research skill, and focusing). All the students in each class presented evidence regarding knowledge, past experience, and action/intervention plan. Specifically, students gained complex management skills in an overthinking session, learned how to concentrate and manage procrastination in a focusing session, skills on becoming an effective teacher, and skills on how to communicate in a class. This signifies that incorporating thematic instruction in the EAC classroom fosters Ph.D. competencies of the students.

**TABLE 1:** Assessment of Ph.D. competencies acquisition using selected academic themes with their corresponding Durette et al. (2016) Ph.D. competence.

Durette et al. (2016) Ph.D. Cluster/competence	Themes	Knowledge	Past experience	Action/intervention plan
Transferable competencies that cannot be formalized/complex management skill	Overthinking	It is described in terms of its causative effect such as mental breakdown	Experience shared is centered on how it affects them during exam preparation	Several strategies to manage mental breakdown include: Studying ahead of time for an exam Identifying the best place and hour to get inspiration Relaxing one’s mind in between studying activities Refocusing one’s mind to get a clear head while pondering on the issue Jotting down ideas when they strikes
Transferable competencies that can be formalized/communication skills	Public speaking	It is based on how the skill can be acquired It is based on how to present at webinars and seminars	Panic attack Taking pills to stay confidence Rehearsing in front of mirror A webinar is less challenging than a seminar	Seeking professional help Getting feedback from a family member before presenting Confronting fear by exposing one’s self to his or her fear gradually
Transferable competencies that can be formalized/innovation management	Research funding	Established the importance of funding research in pure science and humanities equally Local and international Research funding organizations were identified	How and where they received funding for the research is shared How to do a postgraduate program without funding was shared	Plans on how to apply for further studies was shared
Knowledge and technical skills/Teaching	Effective teaching	Teaching is a collaborative activity between a teacher and students Teachers need to be trained in the art of imparting knowledge A good relationship with the students	Experience is related to the difference between local and foreign teachers. Foreign teachers are described to be less particular about an exam	All express what they would like to teach in the future The best way to be an effective teacher is to connect with the student. Commonly called horizontal relationship
Dispositions/Focusing	Focusing	It is described in terms of how to manage procrastination Procrastination is described negatively when an individual chooses not to do what he or she is supposed to do at the right time. On the other hand, it is positive when it is an act aimed at focusing on a task effectively	Experienced shared is about tools used to stay focus on a task especially studying Identification of people and things that serves as distractors while studying	Having short and long time goals by writing them down Taking time to rest before studying Self-knowledge of what works in terms of focusing Having the philosophy of finishing what you start
Knowledge and technical skills/Research skill	Research skill	Students explain their research topics	Reference to research motivation and research methodology The success of past publications Role model in research Views on research ethics An effective method for data collection	Plan to collaborate in terms of data collection Students explain the plan for completing their Ph.D. program

To further establish EAC’s efficacy in developing the students’ skills, students’ perception toward the course was evaluated at the end of the course. The following positive attitudes expressed are as follows.

### Discussion Facilitator

The vast majority of the interviewees commented on the benefits of the video. They all agree that the show provides a good background and context for every assigned topic of discussion. It also helps them to understand academics’ lives, and it motivates them to speak with their partner because they can relate to the video clips. As one interviewee said, *“I improve my communication skills and learn how to talk about different areas of research with appropriate language.”* Another interviewee said, *“the video gives us an attractive perspective about the academic topic of discussion.”*

### Bridging of Gaps

All the students are from different areas of studies. The academic discussion helps them to bridge the gap of information, knowledge, and experiences. The information gap is filled during a *research funding* topic (third week). A doctoral student who possesses the national scholarship provides information to her partner on how to win the scholarship to complete his doctoral program. With respect to knowledge, some of the students share their knowledge based on their expertise in their respective fields. For example, during the topic of *focusing*, a psychologist expert offers suggestions on the best approach to concentrate. Regarding the experiential aspect, every topic of discussion is operationalized by students sharing their personal experiences. All these experiences range from personal and professional experience, and it is shared as a form of information or knowledge for others to learn. All these filled gaps then lead to a research network and collaboration. Students were seen sharing contacts for future research collaboration.

### Fostering of Competencies

The acquisition of the Ph.D. competencies is a function of learners’ ability to express knowledge of the skills, past experience related to the skills, and willingness to continue to build their understanding of the competencies. Some participants appreciate the show’s video clips by establishing that the show is more than entertainment but reflects the life of academics. One participant commented, *“because it allows me to connect with the everyday life of academics, and it also allows me to learn “in some way” about the vicissitudes of academia and research development. But, I don’t know if it’s the same in the social area. I think it is. Perhaps if it is similar to the way of seeing the world from the academic perspective.”* The majority express their willingness to continue watching the show after the course is over. As one respondent indicated, *“Yes, I would love to continue to watch because I really enjoy the story of the characters and how they improve their social skills.”* Also Sheldon, being the main character of the show, is used to express competencies they have acquired in the course. It is interesting to know that the students can identify the character’s weakness and strength in term of competencies they are supposed to acquire. As one interviewee said “*I think Sheldon needs to improve his social skills, but he really gets better at it in the final seasons of the show. Also, he is really smart, but not good at communicating the knowledge to others, and that is not helpful for a young scientist.”*

### Constraints and Feedback

A significant number of the participants expressed the drawbacks of the course. A comment on the time management says “*More time to speak about our ideas and a break at mid-class*.” There is also a comment on giving complete feedback during a discussion. One interviewee says *“From time to time, make suggestions on how to put together the ideas one is expressing. Say the complete sentence”* Another comment is based on the preference of the authentic video as a comprehensible input “*I would like to suggest that the episodes of TTBT should be in English and not in Spanish, so we can actually listen to the phrases that the characters use.”*

### Language Focus

The students believe that the course does not only develop academic cultural competencies, but also has a positive impact on their English speaking ability in different ways. Some believe the conversation will help them in the future when they need to make a presentation in English, one person noting “*I learnt words and academic phrases and skills to make an academic presentation.”* Some believe it is good for their fluency “*This course always involve talking, and that increased my understanding as I could read the context, and also helped me to increase my fluency.*” Some participants make a case for research vocabulary, such as *“I improved my communication skills, and learned how to talk about different areas of research with appropriate language.”*

## Discussion

The purpose of this study was to identify academic themes in TBBT by using Durette et al.’s (2016) Ph.D. reference framework and also, adopting selected identified themes to develop postgraduate students’ Ph.D. competencies.

To address research question 1, the Durette et al. (2016) competency framework is used to identify academic cultural themes in TBBT. All the identified themes are represented in all six clusters of the competency framework. Thus, the usage of the sitcom as a teaching and reflective tool for developing Ph.D. competencies is valid and justified because it demonstrates all the personal and professional competencies in the reference framework. The show also allows the researcher to improve the framework by updating it with competencies such as teaching, impostor syndrome, categorization in academia, and scientific productivity through research publication. All these new competencies address issues that are germane to the new generation of scientists in academia. The positive result is in line with previous studies on TV show’s role as a medium to portray society’s reality. A previous study has focused on teacher representation in movies (Ryan and Townsend, 2010), and this study extends that to a television series.

To address research question 2, a few identified academic cultural themes in TBBT were selected and used to facilitate an EAC course to foster Ph.D. competence. The goal is to test the model of thematic instruction’s efficacy driven by the selected video clips of academic cultural themes from TBBT. The acquisition of the competencies is assessed based on learners’ ability to express knowledge, past experience, and action plans created during and after the course. The results demonstrated that all the skills considered in the course were acquired. In addition, the result shows that the students have positive attitudes toward the course methodology and the sitcom by expressing how the course helps them to be aware of academic cultural competencies and their intention to continue watching TBBT show for entertainment and academic purposes. These findings support the effectiveness of the adopted EAC, which is based on thematic instructions driven by video clips in the TBBT. This confirms the relevance of pop culture’s role in stimulating emotions and providing the context needed for reflection to facilitate effective thematic learning (Blasco et al., 2015).

Another important finding was the efficacy of the EAC course in facilitating the integration of critical thematic video clips for reflective discussion in order to develop learners’ Ph.D. competencies. This can be attributed to the study’s use of a variety of theoretical concepts. First, thematic learning encourages students to take an active role in the classroom by contributing to knowledge construction, sharing experiences, and developing action/intervention plans related to the theme. This finding reaffirms the concept’s beneficial role in fostering an active, interesting, and meaningful learning environment (Min et al., 2012). Second, the importance of dialogic learning argues for the use of discussion to facilitate reflection. It is predicated on the value of discussion to facilitate the exchange of ideas (Haneda, 2017). This is demonstrated by the way students share and bridge gaps in their information, knowledge, and experience. Additionally, the integrated video clips assist in providing context for the discussion, ensuring that participants understand the context of the topic and can relate personally to it. The participants’ appreciated the show’s video clips, which they value because they can relate to the context depicted in the clips as scholars. This demonstrates the critical role of cognitive theory in multimedia learning (Mayer and Moreno, 1998). Finally, reflective practice is used to assess competency acquisition. The evidence of acquisition occurred during the discussion and reflective session when all learners critically shared their personal knowledge, experience, and action plan related to a theme. This finding is consistent with published studies that used similar practices to foster professional competence (Lalor et al., 2015), literacy competence (McLean, 2017), and diagnostic competence (Mamede et al., 2012).

The development of a teaching model for developing academic cultural competencies is critical to a strong doctoral education program. Prior research has highlighted challenges to fostering these competencies (Niemczyk, 2018). This study’s findings show that the starting point for successful implementation of a competency-based doctoral program is to institutionalize and formalize all clusters of academic cultural competencies. This is evident in the positive impact of the EAC course on learners’ outcomes. The justification and adoption of an institutionalized approach is based on the need to have a global and common standard of doctoral education, which currently differs from one national curriculum to another (Nerad and Heggelund, 2011) and varies in learning outcomes due to different modes of doctoral education, such as on-campus and distance modes (Lindner et al., 2001). In addition, unlike the suggestion of movement of researchers through institutions/nations and encouraging graduate student mobility (conferences, student exchanges, seminars, and joint and exchange programs) as indicated in Niemczyk (2018), this study has proffered a cost-effective doctoral-competency training approach. In sum, the findings in this study provide evidence that successful acquisition of Ph.D. competencies involve the use of reflective discussions (Fischer and Zigmond, 1998).

The teaching model’s effectiveness and implementation in this study can be easily institutionalized in several contexts. This is because the adoption of the EAC course is based on the importance of English in academia in terms of productivity and knowledge dissemination. To that end, doctoral programs in countries where English is learned as a Foreign Language can adopt this teaching model to foster research communication in English and Ph.D. competencies. For example, all of the participants’ commented on their improved English oral fluency during the course. Based on English proficiency that might vary among the students, an instructor interested in implementing this teaching model might decide whether the authentic video clips in TBBT should be introduced in English or the learners’ native language. This is because comprehensible input is critical in the context of the competence that is being taught.

With respect to pedagogies in this model, identification of appropriate themes in pop culture to facilitate thematic instruction plays a key role in developing learners’ Ph.D. competency acquisition. The learners can construct each competency knowledge through discussion and acquire the competencies by reflecting on the conversation. This is in line with the recommendation of Niemczyk (2018) on the need to develop researchers’ competency through films and discussions in courses. The success of this model for learning outcomes can also be attributed to the active learning approach integrated into the course design. For example, all the students are actively and experientially involved in the learning process that fills in the information, knowledge, and experiential gaps. The successful bridging of these gaps leads to academic collaboration and networking among the students in the class. Lastly, considering the plethora of identified academic cultural themes with their corresponding competencies in the reference framework, it is nearly impossible to cover all the competencies in the EAC course. Thus, academic cultural competencies can be seen as lifelong learning. That is, a form of learning that learners perform throughout their lives to improve their knowledge, skills, and Ph.D. competencies for personal, societal, or employment-related motives and that occurs outside and after a doctoral program (Field, 2001). Therefore, the TBBT academic cultural competency Supplementary Appendix Table A can be adopted for personal use. It can also serve as a guiding tool for all graduate programs that would like to implement EAC for the purpose of fostering certain Ph.D. competencies among their students.

### Limitations and Conclusion

The findings in this report are subject to at least two limitations. Firstly, few participants are used to assess TBBT videos’ impacts on students’ competency acquisition. This limitation means that the findings need to be interpreted cautiously. The second limitation is that the TBBT academic cultural competencies Supplementary Appendix Table A does not demonstrate all the competencies in the disposition and behavior cluster. Thus, further work needs to be carried out in several contexts to identify more academic cultural themes depicted in TBBT. This can be used to promote philosophical reflections critical to fostering the academic cultural competencies of doctoral students in all higher educational institutions.

The current study’s main goal was to identify academic themes in TBBT using the Durette et al. (2016) Ph.D. reference framework and use the themes to foster doctoral students’ academic cultural competencies in EAC classes. One of the most significant findings is that the show depicts thirty-four competencies across the six clusters of the Durette et al. (2016) reference framework. Integrating several video clips into EAC reveals that the authentic video themes help foster postgraduate students’ academic cultural competencies, which are also known as Ph.D. competencies. This is a scholarly response to a call to create awareness about the academic cultural competencies lacking in many higher educational institutions (Grivillers et al., 2010). One of the key suggestions includes developing researchers’ competencies through films and discussions in courses (Niemczyk, 2018). As a result, this study sheds light on academics’ personal and professional characteristics in popular culture. Concerning research contribution, this research adopts a thematic learning model driven by authentic videos to facilitate and foster Ph.D. competencies. This study’s overall implication in higher education indicates that TBBT is an efficient reflective tool that can be used to develop doctoral students’ competencies in all doctoral training programs. At the individual level, the designed table of TBBT academic cultural competence can be used as a lifelong learning tool that can guide developing competencies among doctoral students, early-career researchers, and senior researchers.

## Data Availability Statement

The raw data supporting the conclusions of this article will be made available by the authors, without undue reservation.

## Ethics Statement

Ethical review and approval was not required for the study on human participants in accordance with the local legislation and institutional requirements. The patients/participants provided their written informed consent to participate in this study.

## Author Contributions

The researcher conceived of the topic, developed the theory, collected the data, verified the analytical methods, and discussed the results and conclusion of the manuscript. The sole author contributed to the article and approved the submitted version.

## Conflict of Interest

The author declares that the research was conducted in the absence of any commercial or financial relationships that could be construed as a potential conflict of interest.

## Publisher’s Note

All claims expressed in this article are solely those of the authors and do not necessarily represent those of their affiliated organizations, or those of the publisher, the editors and the reviewers. Any product that may be evaluated in this article, or claim that may be made by its manufacturer, is not guaranteed or endorsed by the publisher.

## References

[B1] Annisa I. A. (2016). *The Analysis of Interruptions in American Sitcom “The Big Bang Theory” and Its Application in Teaching Speaking.* Doctoral dissertation. Purworejo: Universitas Muhammadiyah Purworejo.

[B2] Arampatzis A. van der Weide T. P. van Bommel P. Koster C. H. A. (2000). “Linguistically-motivated information retrieval,” in *Encyclopedia of Library and Information Science*, ed. Kent A. (New York, NY: Marcel Dekker), 69.

[B3] Arguel A. Jamet E. (2009). Using video and static pictures to improve learning of procedural contents. *Comput. Hum. Behav.* 25 354–359. 10.1016/j.chb.2008.12.014

[B4] Berelson B. (1952). *Content Analysis in Communications Research.* New York, NY: Free Press.

[B5] Berg B. (1989). *Qualitative Research Methods for the Social Sciences.* Toronto: Allyn & Bacon.

[B6] Berk R. A. (2009). Multimedia teaching with video clips: TV, movies, YouTube, and mtvU in the college classroom. *Int. J. Technol. Teach. Learn.* 5 1–21.

[B7] Blasco P. G. Moreto G. Blasco M. G. Levites M. R. Janaudis M. A. (2015). Education through movies: improving teaching skills and fostering reflection among students and teachers. *J. Learn. Through Arts* 11 1–18.

[B8] Bogle D. Dron M. Eggermont J. van Henten J. W. (2010). Doctoral degrees beyond 2010: training talented researchers for society. *Procedia-Soc. Behav. Sci.* 13 35–49. 10.1016/j.sbspro.2011.03.003

[B9] Boud D. Keogh R. Walker D. (1985). *Reflection: Turning Experience into Learning.* New York, NY: Kogan Page/Nichols.

[B10] Bozeman B. (2000). Technology transfer and public policy: a review of research and theory. *Res. Policy* 29 627–655.

[B11] Brick J. (2020). *Academic Culture: A Student’s Guide to Studying at University.* London: Palgrave Macmillan.

[B12] Burant T. J. Gray C. Ndaw E. McKinney-Keys V. Allen G. (2007). The Rhythms of a teacher research group. *Multicult. Perspect.* 9 10–18. 10.1080/15210960701333674

[B13] Campbell J. L. Quincy C. Osserman J. Pedersen O. K. (2013). Coding in-depth semistructured interviews: problems of unitization and intercoder reliability and agreement. *Sociol. Methods Res.* 42 294–320. 10.1177/0049124113500475

[B14] Campinha-Bacote J. (2002). The process of cultural competence in the delivery of healthcare services: a model of care. *J. Transcult. Nursing* 13 181–184. 10.1177/10459602013003003 12113146

[B15] Chelliah K. K. Arumugam Z. (2012). Does reflective practice enhance clinical competency in Medical imaging undergraduates? *Procedia-Soc. Behav. Sci.* 60 73–77. 10.1016/j.sbspro.2012.09.349

[B16] Clarke J. N. Binns J. (2006). The portrayal of heart disease in mass print magazines, 1991—2001. *Health Commun.* 19 39–48. 10.1207/s15327027hc1901_5 16519591

[B17] Collins H. (2010). *Creative Research The Theory and Practice of Research for the Creative Industries.* Singapore: Singapore AVA Publications.

[B18] Creswell J. W. (2014). *Research Design: Qualitative, Quantitative and Mixed Methods Approaches*, 4th Edn. London: Sage Publications Ltd.

[B19] Davies M. Shankar-Brown R. (2011). A programmatic approach to teaming and thematic instruction. *North Carolina Middle School Assoc. J.* 26 1–17. 10.4135/9781452218465.n1 33782627

[B20] Durette B. Fournier M. Lafon M. (2016). The core competencies of PhDs. *Stud. High. Educ.* 41 1355–1370. 10.1080/03075079.2014.968540

[B21] Eggly S. Tkatch R. Penner L. A. Mabunda L. Hudson J. Chapman R. (2013). Development of a question prompt list as a communication intervention to reduce racial disparities in cancer treatment. *J. Cancer Educ.* 28 282–289. 10.1007/s13187-013-0456-2 23440665 PMC3665702

[B22] Etzkowitz H. Kemelgor C. Neuschatz M. Uzzi B. (1994). *Barriers to Women in Academic Science and Engineering. Who Will Do Science.* Baltimore: Johns Hopkins University Press.

[B23] Field J. (2001). Lifelong education. *Int. J. Lifelong Educ.* 20 3–15.

[B24] Fischer B. A. Zigmond M. J. (1998). Survival skills for graduate school and beyond. *New Direct. Higher Educ.* 1998 29–40. 10.1002/9781118667651.ch4

[B25] Flecha R. (2000). *Sharing Words: Theory and Practice of Dialogic Learning.* Lanham, MD: Rowman & Littlefield.

[B26] Follert J. (2015). *Durham Professor Brings Psychology to Life With The Big Bang Theory TV Show.* Available online at: https://www.durhamregion.com/news-story/5612651-durham-professor-brings-psychology-to-life-with-the-big-bang-theory-tv-show/ (accessed August 18, 2021).

[B27] Fraenkel J. R. Wallen N. E. (2009). *The Nature of Qualitative Research. How to Design and Evaluate Research in Education* 7th Edn. Boston, MA: McGraw-Hill 420.

[B28] García-Carrión R. López de Aguileta G. Padrós M. Ramis-Salas M. (2020). Implications for social impact of dialogic teaching and learning. *Front. Psychol.* 11:140. 10.3389/fpsyg.2020.00140 32116941 PMC7012899

[B29] Geerling W. Mateer G. D. Smith B. O. Tierney J. E. Wooten J. J. (2018). *Lesson Plans for Teaching Economics with The Big Bang Theory.* Omaha: University of Nebraska, 163.

[B30] Grivillers E. Lesenne S. Romo M. (2010). *La place des Diplômés d’un Doctorat dans les Entreprises et Les Organismes non Marchands, Rapport D’étude[The Place of Doctoral Graduates in Companies and non-Profit organizations, Study Report]. En ligne, Consulté le 05/11/2017.* Available online at: http://pro-doc.org/fileadmin/doc/pdf/InterReg_Rapport_Pro-Doc.pdf (accessed May 11, 2017).

[B31] Haneda M. (2017). Dialogic learning and teaching across diverse contexts: promises and challenges. *Lang. Educ.* 31 1–5. 10.1080/09500782.2016.1230128

[B32] Hawley K. (2019). I—What is impostor syndrome? *Aristotelian Soc. Suppl. Vol*. 93 203–226. 10.1093/arisup/akz003

[B33] Hewitt A. (2009). *Making a ‘Big Bang’ on TV: 10 questions with David Saltzberg.* Available online at: http://newsroom.ucla.edu/stories/making-a-big-bang-on-tv-10-questions-83027 (accessed March 31, 2011).

[B34] Johns C. (1995). Framing learning through reflection within Carper’s fundamental ways of knowing in nursing. *J. Adv. Nursing* 22 226–234. 10.1046/j.1365-2648.1995.22020226.x 7593941

[B35] Kember D. Leung D. Y. Jones A. Loke A. Y. McKay J. Sinclair K. (2000). Development of a questionnaire to measure the level of reflective thinking. *Assessment Eval. High. Educ.* 25 381–395. 10.1080/713611442

[B36] Kohnke L. Moorhouse B. L. (2020). Facilitating synchronous online language learning through zoom. *RELC J.* 1–6. 10.1177/0033688220937235

[B37] Konus E. (2020). *Using Sitcoms in ESL/EFL: A Handbook for Using Friends in the Classroom. Master’s dissertation.* San Francisco, CA: The University of San Francisco.

[B38] Korek K. (2011). *Big Bang Theory - Conditioning Penny.* Available online at: http://teachinghighschoolpsychology.blogspot.com.au/2011/04/big-bang-theory-conditioning-penny.html (accessed September 12, 2015).

[B39] Krippendorf K. (1980). *Content analysis: An Introduction to Its Methodology.* Thousand Oaks, CA: Sage Publications.

[B40] Krueger R. A. Casey M. A. (2000). *Focus Groups: A Practical Guide for Applied Research.* Thousand Oaks, CA: Sage Publications.

[B41] Lacko H. S. (2011). *Examining Grey’s Anatomy: A Content Analysis of Elements of Medical School Communication Reform in a Popular Medical Drama.* Winston-Salem, NC: Wake Forest University. Doctoral dissertation.

[B42] Lalor J. Lorenzi F. Justin R. A. M. I. (2015). Developing professional competence through assessment: constructivist and reflective practice in teacher-training. *Eurasian J. Educ. Res.* 58 45–66.

[B43] Larkin K. T. Morris T. L. (2015). The process of competency acquisition during doctoral training. *Train. Educ. Professional Psychol.* 9:300. 10.1037/tep0000091

[B44] Lasekan O. Moraga A. Galvez A. (2020). Online marketing by private english tutors in chile: a content analysis of a tutor listing website. *Int. J. Learn. Teach. Educ. Res.* 18 46–62.

[B45] Lee Y. J. (2016). Is it necessary to categorize culture in an L2 learning context? With reference to the American TV sitcom ‘The big bang theory’. *STEM J.* 17 59–76. 10.16875/stem.2016.17.4.59

[B46] Lewis D. Virden T. Hutchings P. S. Bhargava R. (2011). Competence assessment integrating reflective practice in a professional psychology program. *J. Scholarship Teach. Learn.* 11 86–106.

[B47] Li P. Y. R. (2016). *Communicating Science Through Entertainment Television: How the Sitcom The Big Bang Theory Influences Audience Perceptions of Science and Scientists.* Canberra: Australian National University.

[B48] Lin M. T. P. Jain D. J. (2018). Reflective practice: an approach to developing self-knowledge. *Paper Presented at the 11th Taylor’s Teaching & Learning Conference*. “Transforming Curriculum: Empowering Learning for Life”, Subang Jaya.

[B49] Lindner J. R. Dooley K. E. Murphy T. H. (2001). Differences in competencies between doctoral students on-campus and at a distance. *Am. J. Distance Educ.* 15 25–40. 10.1080/08923640109527082

[B50] Lombardi J. (2008). To portfolio or not to portfolio: helpful or hyped? *College Teach.* 56 7–10. 10.3200/ctch.56.1.770

[B51] Lutz G. Roling G. Berger B. Edelhäuser F. Scheffer C. (2016). Reflective practice and its role in facilitating creative responses to dilemmas within clinical communication-a qualitative analysis. *BMC Med. Educ.* 16:301. 10.1186/s12909-016-0823-x 27881123 PMC5121969

[B52] Lynch J. Clinton S. (2020). *NHS Choices. Key Characteristics of Good Competency Evidence.* Available online at: https://nshcs.hee.nhs.uk/knowledgebase/stp-trainee-induction-webinar2-workbased-training-and-assessment/key-characteristics-of-good-competency-evidence/ (accessed August 19, 2021).

[B53] Mamede S. van Gog T. Moura A. S. de Faria R. M. Peixoto J. M. Rikers R. M. (2012). Reflection as a strategy to foster medical students’ acquisition of diagnostic competence. *Med. Educ.* 46 464–472. 22515754 10.1111/j.1365-2923.2012.04217.x

[B54] Manganello J. Franzini A. Jordan A. (2008). Sampling television programs for content analysis of sex on TV: How many episodes are enough? *J. Sex Res.* 45 9–16. 18321026 10.1080/00224490701629514

[B55] Mayer R. E. Moreno R. (1998). A cognitive theory of multimedia learning: implications for design principles. *J. Educ. Psychol.* 91 358–368.

[B56] McHugh M. L. (2012). Interrater reliability: the kappa statistic. *Biochem. Med.* 22 276–282. 10.11613/bm.2012.031 PMC3900052 23092060

[B57] McLean K. (2017). “Using reflective practice to foster confidence and competence to teach literacy in primary schools,” in *Reflective Theory and Practice in Teacher Education*, eds Brandenburg R. Glasswell K. Jones M. Ryan J. (The Gateway East: Springer), 119–139.

[B58] Min K. C. Rashid A. M. Nazri M. I. (2012). Teachers understanding and practice towards thematic approach in teaching integrated living skills (ILS) in Malaysia. *Int. J. Hum. Soc. Sci.* 2 273–281.

[B59] Mirjalili F. Jabbari A. A. Rezai M. J. (2012). The effect of semantic and thematic clustering of words on Iranians Vocabulary learning. *Am. Int. J. Contemporary Res.* 2 214–222.

[B60] Nerad M. Heggelund M. (eds) (2011). *Toward a Global PhD?: Forces and Forms in Doctoral Education Worldwide.* Washington, WA: University of Washington Press.

[B61] Neuman W. L. (2000). *Social Research Methods: Qualitative and Quantitative Approaches*, 4th Edn. Toronto: Allyn & Bacon.

[B62] Niemczyk E. K. (2018). Developing globally competent researchers: an international perspective. *South Afr. J. High. Educ.* 32 171–185.

[B63] Palu I. (2016). *Developing Oral Communication Skills in English Through Sitcoms (The Big Bang Theory).* Tartu: University of Tartu.

[B64] Patton M. Q. (2015). *Qualitative Research and Methods: Integrating Theory and Practice.* Thousand Oaks, CA: Manganello.

[B65] Plack M. M. Driscoll M. Blissett S. McKenna R. Plack T. P. (2005). A method for assessing reflective journal writing. *J. Allied Health* 34 199–208. 16529182

[B66] Rees G. (2005). *Find the Gap–Increasing Speaking in Class.* Available online at www.teachingenglish.org.uk/article/find-gap-increasing-speaking-class (accessed December 27, 2020).

[B67] Ryan P. A. Townsend J. S. (2010). Representations of teachers’ and students’ inquiry in 1950s television and film. *Educ. Stud.* 46 44–66.

[B68] Shen X. Tian X. (2012). Academic culture and campus culture of universities. *High. Educ. Stud.* 2 61–65.

[B69] Strauss A. Corbin J. (1998). *Basics of Qualitative Research: Techniques and Procedures for Developing Grounded Theory.* Thousand Oaks, CA: Sage.

[B70] Tan E. S. (2018). A psychology of the film. *Palgr. Commun.* 4 1–20.

[B71] Teo P. (2019). Teaching for the 21st century: a case for dialogic pedagogy. *Learn. Culture Soc. Interact.* 21 170–178. 10.1016/j.lcsi.2019.03.009

[B72] Thomas N. (2010). *Making a Big Bang on the Small Screen.* Available online at: https://physicsworld.com/a/making-a-big-bang-on-the-small-screen/ (accessed August, 19, 2021).

[B73] Tierney J. Dirk M. Geerling W. Wooten J. Smith B. (2015). *Bazinganomics: Economics of The Big Bang Theory.* Omaha: University of Nebraska.

[B74] Verderame M. F. Freedman V. H. Kozlowski L. M. McCormack W. T. (2018). Point of view: competency-based assessment for the training of PhD students and early-career scientists. *Elife* 7:e34801. 10.7554/eLife.34801 PMC6002247 29848440

[B75] Weitekamp M. A. (2017). The image of scientists in The Big Bang Theory. *Phys. Today* 70 40–48. 10.1063/pt.3.3427

[B76] Ye Y. Ward K. E. (2010). The depiction of illness and related matters in two top-ranked primetime network medical dramas in the United States: a content analysis. *J. Health Commun.* 15 555–570. 10.1080/10810730.2010.492564 20677058

