# Supplementary material for: Identification and Adoption of Themes in The Big Bang Theory Sitcom to Foster Academic Cultural Competencies of Doctoral Students in English for Academic Conversation Classroom
Source: Front Psychol. 2021 Sep 9;12:699662. doi: 10.3389/fpsyg.2021.699662 (PMC8458569; doi:10.3389/fpsyg.2021.699662)
Supplement: Supplementary file 1 [file Table_1.docx]

| Appendix A: TBBT Academic Cultural Themes’ Table | | | | |
| --- | --- | --- | --- | --- |
| Clusters of Ph.D Competency  (Durette et al., 2016) | Competencies | Latent or manifest content analysis | Scene description | Season (S), episode (E) Scene |
| Knowledge and Technical skills | Monodisciplinary | Latent | Sheldon as a theoretical physicist  Leonard as an experimental physicist  Raj as an Astrophysicist  Amy as a Neurobiologist  Bernadette as a microbiologist  Howard as an Engineer | Throughout the seasons |
|  | Pluridisciplinary | Manifest | Sheldon and Amy create an experiment based on a theory of meme | S4, E20 |
|  | Teaching | Manifest | Sheldon gets feedback on his teaching  Sheldon seeks help to improve his teaching skills  Sheldon questions Howard’s capability to teach his class | S4,E14;S3,E10 |
|  | Research Publication | Manifest and latent | Amy tells Sheldon about her published work in ….  Sheldon claims his boss published his research  Sheldon and Leonard disagree on sharing credit for a publication  Race for publication (helium) | S5,E12; S9,E6 |
| Transferable Competencies that can be Formalized | Knowledge of the professional environment, academic environment and industrial environment | Manifest and latent | Howard, Amy, and Leonard’s laboratory  Characters offices  Science bowl event  Award ceremony for Sheldon  The tradition of showing new staff around the campus  Bernadette working in an industrial setting | S12,E17 |
|  | Professional conduct (regulation, ethics and safety) | Manifest and latent | Retraction of published work  Keeping military projects classified  Characters working in the lab using of a portable safety shield  Sheldon using sexist language in a workplace | S9,E6 |
|  | Academic Communication skills | Manifest and latent | Amy and Sheldon on the usage of academic vocabulary in their conversation  Usage of academic vocabulary throughout the show  Sheldon fear of public speaking/communication | S4,E5  S3,E18 |
|  | IT skills | Latent | Sheldon consulting for people in an IT store | S1,E16 |
|  | Innovation management (Scientific watch and  Research valorization) | Manifest and latent | Research funding dinner  Collaboration between university and government agencies  Fish nightlight  Inventing three players chess game | S4,E15 |
|  | Project Management |  | Sheldon, Leonard, and Howard working on a military project | S10,E3 |
|  | Languages | Manifest and Latent | Howard speaks many languages  Howard teaches Sheldon mandarin  All the scientists speak Klingon | S1,E17 |
|  | Commercial skills | Manifest and latent | Sheldon spends 46% of his income  Leonard helps Penny to plan her budget  The scientists decided to invest in Stuart’s comic book store business  Howard plans to create and sell a patent to raise his child | S9.E16 |
|  | Administrative management | Manifest | Sheldon hires an assistant | S3,E5 |
|  | Categorization in academics | Manifest | Sheldon, Raj, and Leonard are competing for a tenure position  Sheldon is promoted to a junior professorship | S8,E2 |
|  | Imposter syndrome | Latent | Amy sheds light on the concept of impostor syndrome that affects most academics | S12,E18 |
| Transferable Competencies that cannot be Formalized | Lateral thinking | Manifest | Sheldon providing solutions to a Middle East issue | S2.,E22 |
|  | Cognitive abilities | Latent | Eidetic memory | S3, E5 |
|  | Complex problems management | Latent | Sheldon manages mental breakdown because of overthinking | S3, E14 |
|  | Ability to collaborate | Latent | The four main characters collaborate on many occasions | S10, E2 |
|  | Leadership | Latent | Leonard’s leadership role | S12,E7 |
|  | Teamwork | Manifest | In the annual physics bowl competition, learning how to be a team player | S1, E13 |
| Dispositions | Focusing | Manifest | The scientists create a technique that will make them focus on a task | S8,E5 |
|  | Curiosity | Manifest | Raj, Howard and Sheldon searching for crickets in order to determine their species | S3, E2 |
|  | Persuasion | Manifest | Sheldon persuades Howard to show his paper to Stephen Hawking | S5,E21 |
|  | Originality | Manifest | Leonard’s work is considered derivative | S3,E10 |
|  | Versatility | Latent | Sheldon’s knowledge in different fields | S3,E22;S1,E4 |
|  | Accuracy | Manifest | Scientific accuracy of Superman theory | S1,E2 |
| Behaviors | Dreamer | Manifest | Sheldon dreams of winning Nobel Laureate someday | S3,E1 |
|  | Hygiene | Manifest and latent | Sheldon asks Raj to explain his sneeze before sitting with him  Sheldon does not like visiting hospitals because of germs  Sheldon uses a baby wipe before having his meal  Sheldon provides Purell to friends before coming to his house  Sheldon expresses his germaphobic attitude | S2,E4;S5,E7 |
|  | Humour | Latent | All the characters in this show have a sense of humour | S1-S12 |
|  | Understanding/tolerance | Manifest | Sheldon admits his friends' tolerance has helped him to achieve success at the Nobel Laureate presentation award | S12,E24 |
|  | Punctuality | Latent | Movie theatre scene | S4, E8 |
|  | Service-minded | Manifest | Inspired young girls in high school to pursue science | S6,E18 |
| Meta-competencies | Ability to learn  and Adaptability | Latent | Sheldon adapts to all the changes that happen in his life. First, Leonard and Penny’s engagement announcement, an accidental fire that razes the comic book store and Sheldon does not want to move from string theory research to inflationary cosmology | S7,E24 |
