# Supplementary material for: Identification and Adoption of Themes in The Big Bang Theory Sitcom to Foster Academic Cultural Competencies of Doctoral Students in English for Academic Conversation Classroom
Source: Front Psychol. 2021 Sep 9;12:699662. doi: 10.3389/fpsyg.2021.699662 (PMC8458569; doi:10.3389/fpsyg.2021.699662)
Supplement: Supplementary file 2 [file Table_2.docx]

Appendix B

Question Prompt List

| N/s | The topic for each class | Question Prompt Lists |
| --- | --- | --- |
| 1. | Teaching | 1. What should Sheldon do to improve his teaching skills? 2. Have you ever taught before? If yes, how was the experience? 3. Do you think high school teachers are better than teachers in universities? 4. Is there any difference between Chilean teachers and foreign teachers? 5. What are the qualities of a good teacher? 6. Do you think all researchers are good at teaching? 7. Do you think teaching is an innate ability? 8. Who is your favourite teacher? And why? 9. Would you rather learn from a young teacher or experienced teacher? 10. If you were going to be a teacher, where and what would you like to teach? |
| 2. | Focusing | 1. What is procrastination? 2. How many hours do you spend on research/studying every day? 3. What kinds of things do you postpone doing? 4. What can procrastinators do to change their lives? 5. Do you ever forget to pay your bills on time? 6. Have you ever forgotten an important date or event? 7. Are you always on time to class, late, or early? 8. Do you want to accomplish many things in your life, or do you want to take it slow? 9. What is the best time of the day for you to study? 10. What always distracts you when you study? 11. What are the strategies you use to keep your mind focused on a task? |
| 3. | Overthinking | 1. Have you ever had a mental breakdown because of overthinking? 2. How do you prepare for an exam? 3. How and where do you get inspiration for your creativity? 4. Do you overthink a lot? 5. What do you think is the best way to be creative? 6. Do you think the topic of your research is difficult? 7. How do you stop overthinking? 8. When is the best time for you to study? 9. Does your research require deep thinking? |
| 4. | Research Funding | 1. Do you need funding for your research? 2. What specifically do you need funding for? 3. What are the research funding organizations you know in the country and abroad? 4. Is your scholarship sufficient for your research? 5. Do you think pure sciences should receive more funding than arts and humanities? 6. How can postgraduate students survive without funding? 7. What is Sheldon’s attitude towards research donors? |
| 5. | Research Skill | 1. Describe your research topic? 2. What is your motivation for choosing this research topic? 3. What type of research methodology are you adopting for your research? 4. How are you going to disseminate your findings? 5. What is your opinion of closed and open access journals? 6. How can you describe your relationship with your supervisor? 7. When do you plan to complete your graduate program? 8. Who do you consider as your mentor in your area of research? 9. What is your research plan? 10. What are the challenges have you faced or do you foresee in your research? 11. What is your opinion on using animals to conduct research? |
| 6. | Public Speaking | 1. What is your experience with public speaking with respect to presentations at conferences? 2. What advice do you have for someone that is afraid to speak in public? 3. Webinar or Seminar? Which one do you prefer? 4. How do you prepare your PowerPoint for your public presentation? 5. Have you made a public presentation in English in the past? |
